# GTPase Era at the heart of ribosome assembly

**DOI:** 10.3389/fmolb.2023.1263433

**Published:** 2023-10-04

**Authors:** Christelle Gruffaz, Alexandre Smirnov

**Affiliations:** ^1^ UMR7156- Génétique Moléculaire, Génomique, Microbiologie (GMGM), University of Strasbourg, Centre National de la Recherche Scientifique (CNRS), Strasbourg, France; ^2^ University of Strasbourg Institute for Advanced Study (USIAS), Strasbourg, France

**Keywords:** ERAL1, GTPase, KH domain, ribosome assembly, bacteria, mitochondria, Era, Perrault syndrome

## Abstract

Ribosome biogenesis is a key process in all organisms. It relies on coordinated work of multiple proteins and RNAs, including an array of assembly factors. Among them, the GTPase Era stands out as an especially deeply conserved protein, critically required for the assembly of bacterial-type ribosomes from *Escherichia coli* to humans. In this review, we bring together and critically analyze a wealth of phylogenetic, biochemical, structural, genetic and physiological data about this extensively studied but still insufficiently understood factor. We do so using a comparative and, wherever possible, synthetic approach, by confronting observations from diverse groups of bacteria and eukaryotic organelles (mitochondria and chloroplasts). The emerging consensus posits that Era intervenes relatively early in the small subunit biogenesis and is essential for the proper shaping of the platform which, in its turn, is a prerequisite for efficient translation. The timing of Era action on the ribosome is defined by its interactions with guanosine nucleotides [GTP, GDP, (p)ppGpp], ribosomal RNA, and likely other factors that trigger or delay its GTPase activity. As a critical nexus of the small subunit biogenesis, Era is subject to sophisticated regulatory mechanisms at the transcriptional, post-transcriptional, and post-translational levels. Failure of these mechanisms or a deficiency in Era function entail dramatic generalized consequences for the protein synthesis and far-reaching, pleiotropic effects on the organism physiology, such as the Perrault syndrome in humans.

## Introduction

All living beings make proteins. Most importantly, they also make the molecular machines that make proteins—ribosomes. Even though the ribosomes themselves are remarkably conserved and shared by all groups of organisms, nature has evolved two divergent ribosome biogenesis paradigms, which can be called ‘*bacterial*’ (including its derived mitochondrial and plastid variants) and ‘*archaeal*’ (comprising the highly evolved eukaryotic ribosome assembly pathway). Albeit conceptually similar, the two assembly programs differ in their mechanisms and molecular players (Shajani et al., 2011; Davis and Williamson, 2017; Bohnsack and Bohnsack, 2019; Klinge and Woolford, 2019; Ferrari et al., 2021; Jüttner and Ferreira-Cerca, 2022). This fundamental molecular divide between ‘bacteria-like’ and ‘archaea-like’ genetic systems is central to our understanding of the basic organizational principles of Life and its evolution, including the origin of eukaryotes which combine both these approaches to ribosome biogenesis within one cell.

Here, we will focus on the biogenesis of the bacterial ribosomal small subunit (SSU); its chloroplast and mitochondrial counterparts are formed in a similar way and mostly with the help of homologous factors (Shajani et al., 2011; Davis and Williamson, 2017; Ferrari et al., 2021). The SSU biogenesis pathway begins co-transcriptionally and involves the sequential folding of the 16S rRNA domains: the 5’-, the central, and the 3′-domains (the latter subdivided into a major and a minor subdomains). Their folding is accompanied—and partially directed—by the binding of ribosomal proteins (r-proteins) and several dozens of maturation factors. The rRNA domains and the associated r-proteins form the body, the platform, and the head of the SSU. The maturation factors transiently bind to the nascent SSU and play diverse roles. RNases cleave the polycistronic rRNA precursor (this step is usually performed by RNase III) and, at later stages, carve the correct 16S rRNA termini (Dunn and Studier, 1973; Bechhofer and Deutscher, 2019). Modification enzymes introduce functionally important chemical changes at specific positions of 16S rRNA and some r-proteins. For example, the universally conserved methyltransferase KsgA/RsmA/Dim1/TFB1M methylates an invariant adenine in the tip of the helix 45 (h45) of the 3′-minor domain, thereby participating in the platform formation (Connolly et al., 2008). Some assembly factors (AFs) do not alter the covalent structure of the SSU constituents; instead, they guide and rhythm the assembly by enforcing correct folding, recruiting r-proteins and other factors, or blocking premature interactions and untimely architectural events (Shajani et al., 2011; Maksimova et al., 2022). For example, the AFs YqeH/NOA1 and RimM orchestrate the assembly of the body and the head, respectively (Loh et al., 2007; He et al., 2012; Guo et al., 2013), whereas Era/ERAL1, RbfA, and RsgA sequentially shape the platform (Schedlbauer et al., 2021; Itoh et al., 2022; Harper et al., 2023). Among these factors, the GTPases YqeH, Era and RsgA are especially interesting, since they can couple GTP hydrolysis to conformation switching, thereby regulating their interactions with the nascent SSU (Britton, 2009; Maiti et al., 2021).

This systematic review is about Era, one of the most widespread and functionally critical ribosome biogenesis factors from the ‘bacterial party’. Athough Era is equally essential in *Escherichia coli* and in our mitochondria, its exact molecular mechanisms remain obscure. A huge amount of structural, biochemical, and genetic data about its organization, activities, interactions, and physiological outreach, accumulated over the last 50 years, offer today a complex mosaic of observations, where a few key themes are only beginning to take shape. The present review focuses on these central themes of Era biology and the ways they are manifested across a variety of ‘bacterial-type’ genetic systems, including bacteria, eukaryotic mitochondria, plant chloroplasts, and even human patients suffering from a specific kind of Era-linked mitochondrial disease.

## Evolutionary journey of Era GTPases

### Origin, spread, and essentiality of Era

Translational factor-related (TRAFAC) GTPases are one of the most ancient protein groups that existed already in the Last Universal Common Ancestor and includes today such fundamental components of any genetic system as, for example, the elongation factors Tu and G. It is within this primordial class of enzymes that, soon after Bacteria have diverged from Archaea, one specific branch has seen a particularly broad—and evolutionarily successful—expansion, spawning such functionally diverse proteins as septins, the iron transporter FeoB, the tRNA modification enzyme TrmE, the ribosomal large subunit (LSU) AFs YihA and EngA/Der, and finally Era (Leipe et al., 2002; Goto et al., 2013). The characteristic fusion of the N-terminal GTPase domain with the C-terminal KH domain created the unique “face” of the Era protein family and sealed its destiny to serve important roles in cellular RNA metabolism.

**FIGURE 1:**
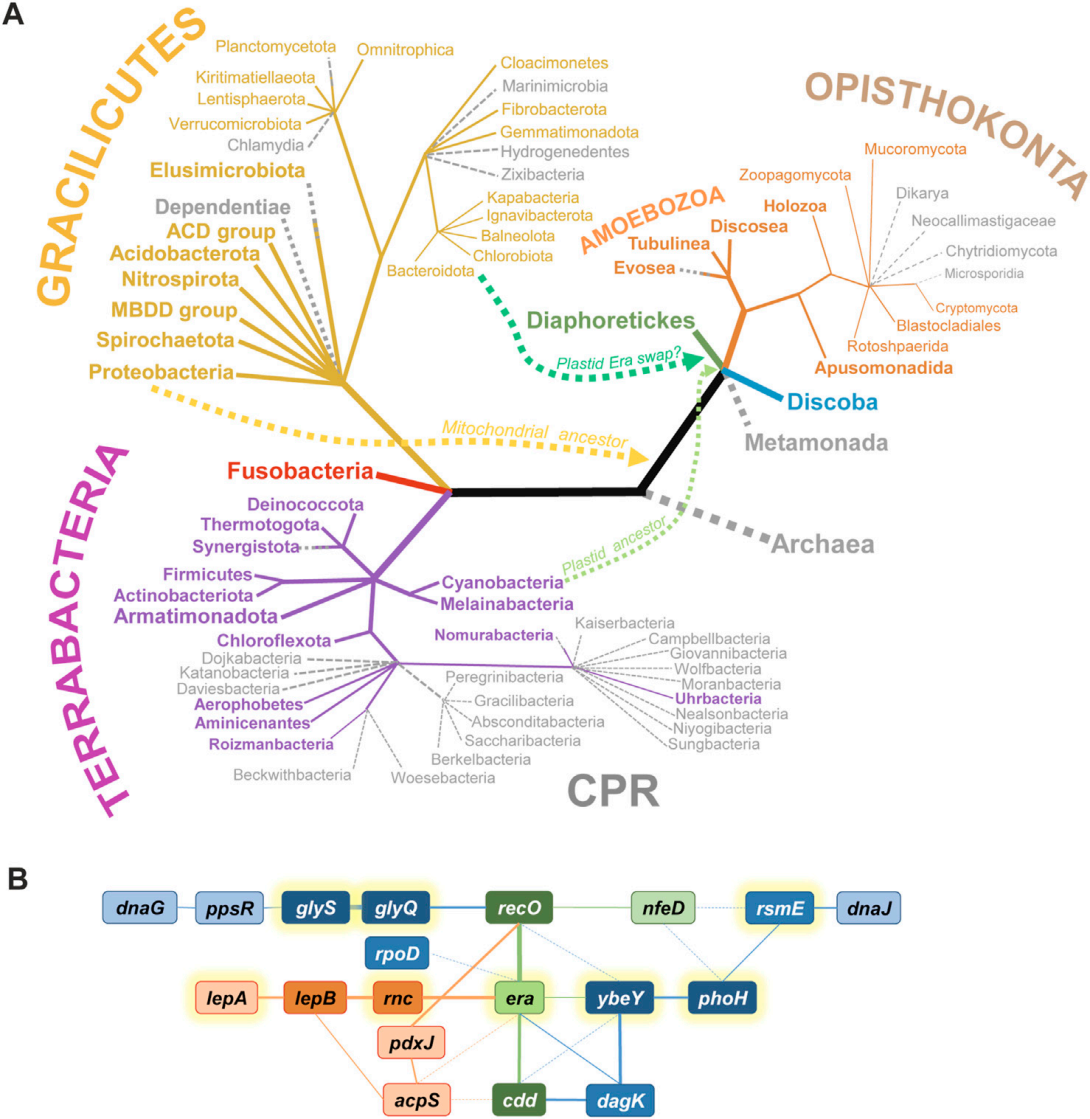
The phylogenetic distribution and the genomic context of *era* genes. **(A)** The presence of *era* across the Tree of Life. Groups possessing an *era* gene are shown in color, while those lacking recognizable Era homologues are shown in grey as dotted branches. Archaea apparently have never had *era*. Gracilicutes and Terrabacteria include classical Gram-negative and Gram-positive bacteria, respectively; most of them possess an Era homologue, with the exception of a few genome-reduced phyla. CPR is the Candidate Phyla Radiation (sister to or part of Chloroflexota, according to most recent reconstructions); most CPR phyla have lost *era*. Among Eukarya, *era* disappeared in non-respiring clades (Metamonada, Microsporidia) and higher fungi. Other Opistokonta, including animals (Holozoa) have an Era homologue. Diaphoretikes is a supergroup including all plastid-containing eukaryotic phyla (Glaucophyta, Rhodophyta, Viridiplantae, Stramenopiles, Alveolata, Rhizaria, Cryptophyta); they all possess at least one Era homologue. Discoba includes Jakobida, Heterolobosea and Euglenozoa; they all seem to have *era*. For some clades (Planctomycetota, Elusimicrobiota, Synergistota, Nomurabacteria, Evosea), *era* was found in some species but not in others. Curvy lines show the migration of *era* genes from ancestors of mitochondria and plastids to early Eukarya. The phylogenetic tree (not in scale) is based on Adl et al., 2019; Coleman et al., 2021; Moody et al., 2022. **(B)** Overview of syntenic groups involving *era* in Gracilicutes (red), Terrabacteria (blue), or both groups (green). Deeper color and thicker lines correspond to more frequent associations. Full lines denote direct neighborhood, dotted lines mean a more distant synteny. *acpS*, holo-[acyl-carrier-protein] synthase; *cdd*, cytidine/deoxycytidine deaminase; *dagK*, diacylglycerol kinase; *dnaG*, DNA primase; *dnaJ*, chaperone protein; *glyQ* and *glyS*, subunits of glycyl-tRNA synthetase; *lepA*, 30S ribosomal subunit biogenesis factor/elongation factor 4; *lepB*, signal peptidase I; *nfeD*, NfeD-like C-terminal domain-containing protein (OB-fold); *pdxJ*, pyridoxine 5′-phosphate synthase; *phoH*, PhoH domain-containing putative ATPase YbeZ/PhoL; *ppsR*, phosphoenolpyruvate synthase regulatory protein; *recO*, DNA repair protein; *rnc*, double-stranded RNA specific endoribonuclease III; *rpoD*, housekeeping sigma-factor σ^70^/σ^A^; *rsmE*, 16S rRNA m^3^U1498 methyltransferase; *ybeY*, uS11-interacting 30S ribosome assembly factor. Genes encoding translation-related proteins are highlighted with yellow halos.

Somehow, Era imposed itself as a critical component of the SSU biogenesis, which must have made it indispensable already in early bacteria and ensured its widest spread and conservation in most extant bacterial phyla (Figure 1A). Indeed, congruent with its early origins, Era is often an essential protein (e.g., in *E. coli, Salmonella enterica, Haemophilus influenzae, Synechococcus elongates*—Inada et al., 1989; Takiff et al., 1989; Lerner and Inouye, 1991; Anderson et al., 1996; Akerley et al., 2002; Voshol et al., 2015). And even when it is not (e.g., in *Staphylococcus aureus, Streptococcus pneumoniae, Mycobacterium tuberculosis,* and some *Bacillus subtilis* strains), the loss of Era is associated with severe pleiotropic phenotypes (Minkovsky et al., 2002; Zalacain et al., 2003; DeJesus et al., 2017; Wood et al., 2019; Bennison et al., 2021). This unacceptably high fitness cost made the secondary loss of Era though not impossible, certainly very difficult. Among the known bacteria, only some extremely genome-reduced groups, such as Dependentiae, Chlamydia, and most Candidate Phyla Radiation (CPR), managed to overcome this “Era addiction” (Leipe et al., 2002; Gil et al., 2004). This trend is paralleled by simplification of their ribosomes, including the shortening of rRNAs and the disappearance of some r-proteins (Tsurumaki et al., 2022).

The next turn in the evolutionary saga of the Era family was the emergence of eukaryotes. While Archaea have never possessed such proteins, the arrival of bacterial endosymbionts—the ancestors of modern mitochondria and plastids—opened the way to acquire Era horizontally. This new evolutionary spread campaign turned out to be extremely successful. Besides obligate anaerobic eukaryotes with degenerated, genome-lacking mitochondria (Metamonada, Microsporidia), only higher fungi have lost Era, while essentially all other major eukaryotic clades (Discoba, the plastid-containing Diaphoretikes, most Amoebozoa and Opisthokonta, including animals) have kept it (Figure 1A). Although in all known Eukarya the *era* genes are now part of nuclear DNA, they encode exclusively mitochondrial or plastid-localized proteins that participate in the maturation of the corresponding organellar ribosomes (Panigrahi et al., 2009; Dennerlein et al., 2010; Uchiumi et al., 2010; Sun et al., 2019; Maiti et al., 2021; Méteignier et al., 2021; Valach et al., 2023). Plants typically have two Era homologues with nonoverlapping localizations in mitochondria and chloroplasts (Ingram et al., 1998; Suwastika et al., 2014; Cheng et al., 2018; Sun et al., 2019).

Intriguingly, while the origin of the plant mitochondrial Era proteins is transparent (their sequences clearly cluster with α-proteobacterial ones), the source of the chloroplast Era is more enigmatic. Instead of grouping with cyanobacterial Era proteins, they show significant similarity with those from the Bacteroidetes-Chlorobi group, suggesting that another horizontal transfer event was responsible for their emergence in the plant kingdom (Suwastika et al., 2014).

Given their critical roles in the organellar gene expression, eukaryotic Era proteins are typically essential for viability (Ingram et al., 1998; Gohda et al., 2003; Cheng et al., 2018), although there might be exceptions (Sun et al., 2019), and cell lines lacking mitochondrial Era, however extremely sick due to the lack of respiration, can be maintained on high-glucose media (Uchiumi et al., 2010; Xie et al., 2012).

Pointing at a deeply conserved biological role, Era homologues show high functional portability. Thus, *S. enterica*, *Pseudomonas aeruginosa*, *Francisella tularensis*, *B. subtilis, Listeria monocytogenes,* and *Streptococcus mutans* Era proteins complement *E. coli era* mutations, while human mitochondrial ERAL1 can replace its chicken counterpart (Pillutla et al., 1995; Anderson et al., 1996; Zuber et al., 1997; Powell et al., 1999; Minkovsky et al., 2002; Gohda et al., 2003; Auvray et al., 2007).

### Synteny of *era* genes

The genomic environment is often informative about the function and the transmission mode of genes, especially in prokaryotes. While the genomic neighborhood of *era* varies across Bacteria, the synteny tends to be relatively well preserved within individual microbial clades. This property suggests primarily vertical spread of this gene among Bacteria, as expected of a highly conserved and functionally critical protein. Indeed, published phylogenetic trees of Era closely follow the established phyletic relationships between grand taxa (Mittenhuber, 2001; Coleman et al., 2021; Moody et al., 2022).

Looking at *era* syntenies across multiple model species, one can single out a few genes that pop up time and again next to *era* or in its close proximity (Figure 1B). Thus, in many Terrabacteria, *era* is adjacent to genes encoding the ribosome assembly factor YbeY and its highly conserved partner YbeZ/PhoH-like, involved in the biogenesis of the platform and the 3′-domain of 16S rRNA (Liao et al., 2021; Andrews and Patrick, 2022). Three other genes associated with ribosome biogenesis and frequently found close to *era* are *rnc, rsmE,* and *lepA*. *rnc* encodes RNase III, which is involved in rRNA pre-processing at earliest stages of ribosome biogenesis (Dunn and Studier, 1973); the *rnc* gene precedes *era* in many species and is translationally coupled to the latter in many γ-proteobacteria (see the section “Post-transcriptional regulation in bacterial *rnc-era* operons”). RsmE is m^3^U1498 methyltransferase that methylates a conserved uridine in the 3′-minor domain of 16S rRNA (Basturea et al., 2006). LepA, another ancient TRAFAC GTPase also known as elongation factor 4, has been shown to participate in the SSU assembly, primarily at the level of the 3′-domain of 16S rRNA (Gibbs et al., 2017). Since the function of Era is intimately linked to the maturation of the platform and the 3′-domain of 16S rRNA, its connection with these proteins is functionally meaningful.

A few other neighbors associate Era more loosely with central genetic processes, such as replication (*dnaG*), recombination (*recO*), transcription (*rpoD*), translation (*glyQS*), and protein folding (*dnaJ*). Finally, some metabolic enzymes are unusually often encoded close to *era*; some of them are also frequent satellites of *ybeY* genes (*cdd*, *dagK*, *pdxJ*, *acpS*). The mechanistic meaning of these persistent syntenic associations with metabolic enzymes remains enigmatic. However, the majority of *era* neighbors belong to a group of most deeply conserved bacterial genes, highlighting the ancient origin and centrality of Era to the cellular metabolism (Gil et al., 2004).

## Structure-function of Era at the molecular level

**FIGURE 2:**
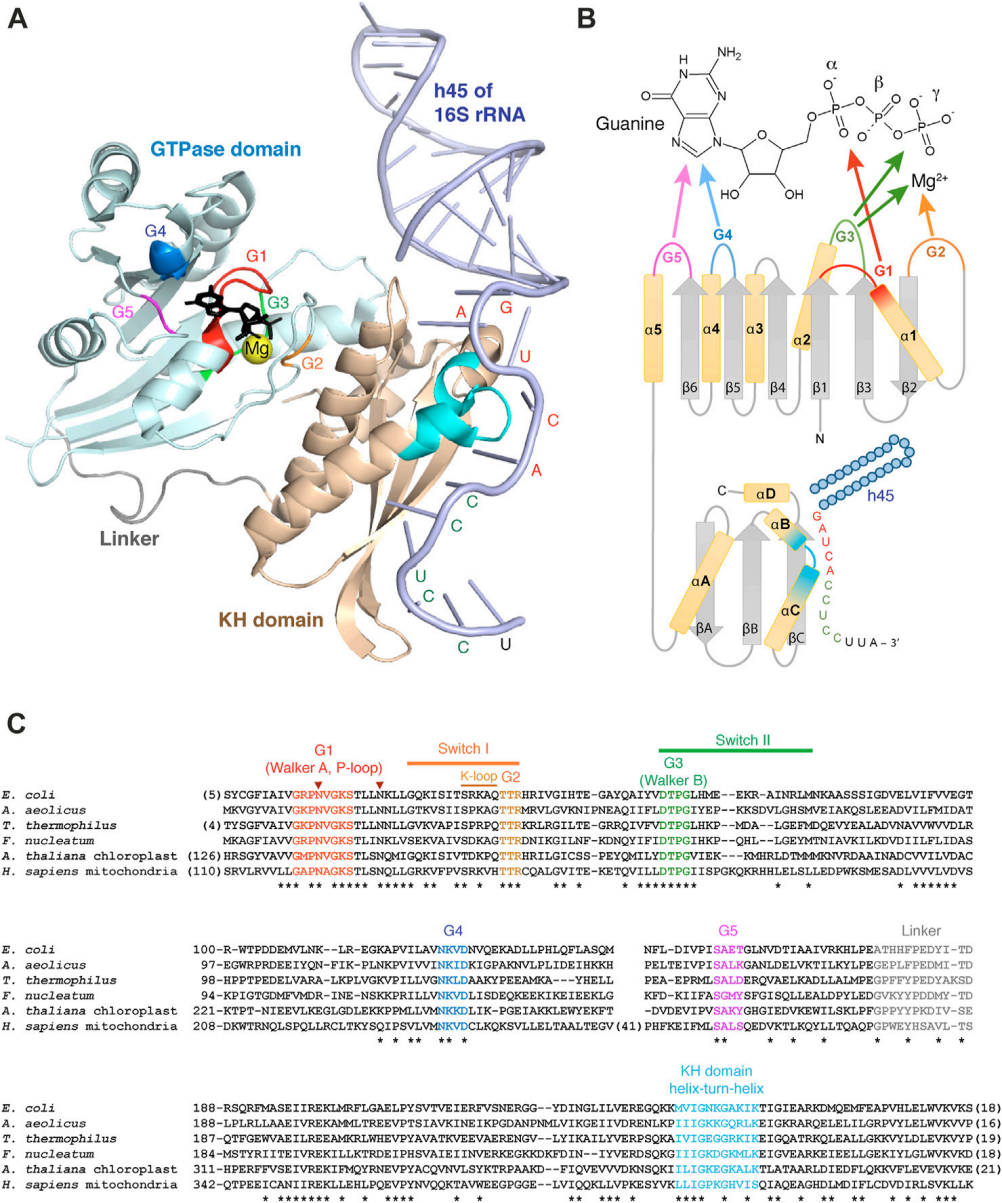
Structural aspects of Era and its interactions with GTP and RNA. **(A)** Crystal structure of *A. aeolicus* Era in complex with GDPNP and h45 of 16S rRNA (3r9x) (Tu et al., 2011). The two Era domains and the linker are shown in different colors; GDPNP is black. The G1-G5 motifs of the GTPase domain and the helix-turn-helix motif of the KH domain are highlighted in the same color code as in the panels **(B, C)**. The nucleotides of the single-stranded 3′-tail of 16S rRNA interacting with Era are labelled in red (GAUCA) or green (anti-SD sequence). **(B)** Secondary structure diagram of Era and its interactions with GTP and h45. **(C)** Multiple sequence alignment of Era homologues from Gracilicutes (*E. coli*, *A. aeolicus*), Terrabacteria (*T. thermophilus*), Fusobacteria (*F. nucleatum*), plant chloroplasts (*A. thaliana*), and animal mitochondria (*H. sapiens*). Conserved positions are tagged with asterisks. The two invariant asparagines involved, together with the K-loop, in K^+^ binding are labelled with arrowheads. The alignment is performed with COBALT (Papadopoulos and Agarwala, 2007).

**FIGURE 3:**
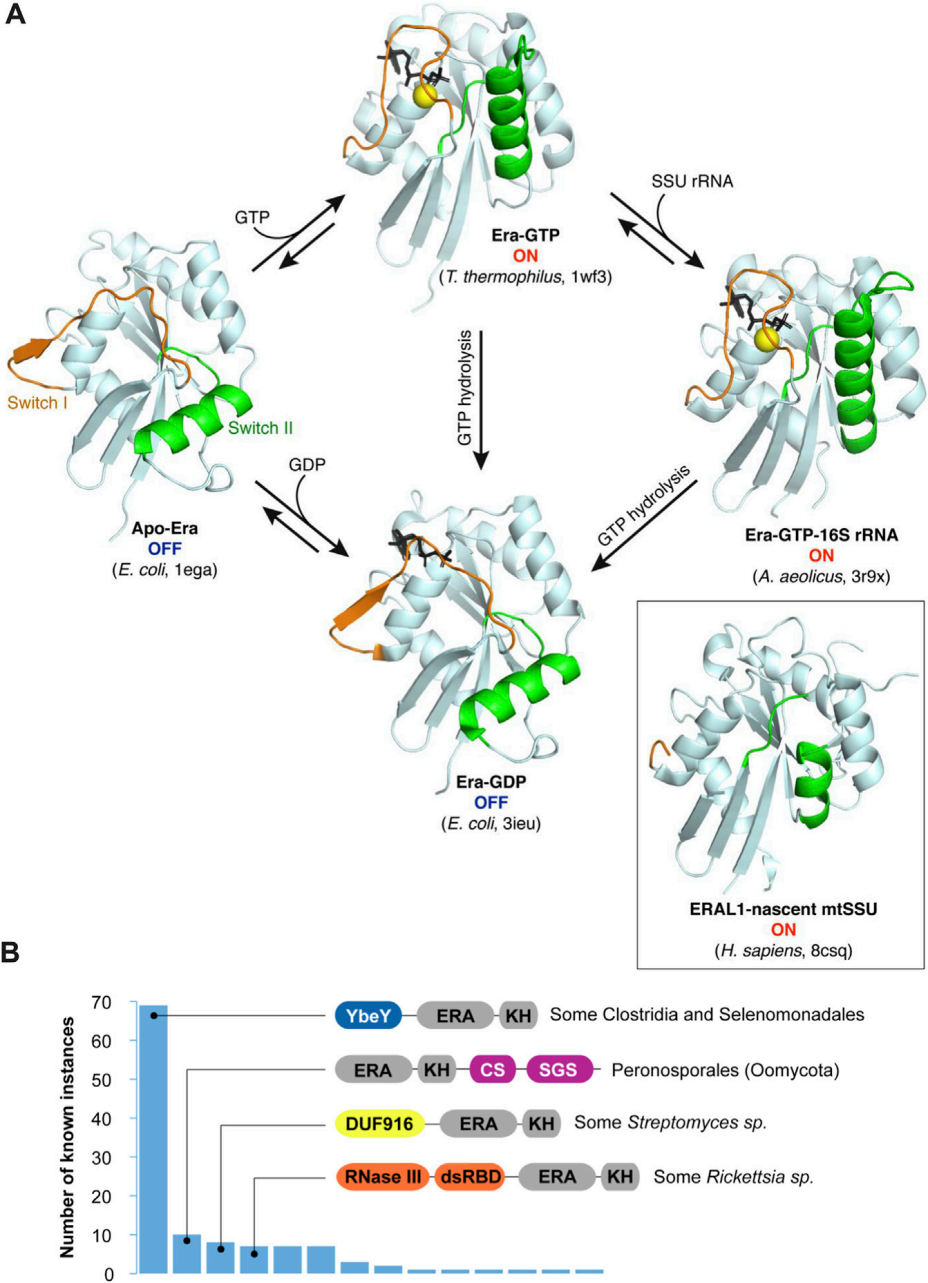
The switching behavior of the G-domain and alternative, multidomain architectures of Era proteins. **(A)** Conformation switching between different Era forms. The GTPase domains of bacterial Era proteins in different binding states are shown in the same orientation to highlight the movement of the switch regions upon GTP binding and hydrolysis (Chen et al., 1999b; Tu et al., 2009; 2011). Guanosine nucleotides are shown in black (“GTP” is represented by its nonhydrolyzable analogue GDPNP). The inset shows the conformation of the mitochondrial Era homologue ERAL1 associated with a nascent SSU (Harper et al., 2023). It lacks nucleotides, and the switches are only partially resolved. However, the overall conformation resembles the ON-state of the bacterial proteins. **(B)** Known fusions of Era with other domains (as queried from InterPro in June 2023—Paysan-Lafosse et al., 2023). Canonical Era consists of two domains shown here in grey: the larger Era-type GTPase domain (“ERA”) and the smaller KH domain (“KH”). However, in some clades, this basic architecture is extended by extra domains, the most prevalent of those being YbeY (IPR002036), CS (IPR007052), SGS (IPR007699), DUF916 (IPR010317), and the two domains forming RNase III (IPR000999 and IPR014720).

Typically, Era proteins are relatively small (300–350 amino acids, ∼35 kDa) and consist of two globular domains connected by an unstructured linker. The N-terminal GTPase domain binds guanosine nucleotides; it likely works as a molecular switch triggered by GTP hydrolysis and reset by GDP/GTP exchange (Bourne et al., 1991; Paduch et al., 2001). The C-terminal KH domain confers RNA-binding activity and is responsible for the association of Era with ribosomes; it is an important diagnostic feature of the Era family of TRAFAC GTPases (Leipe et al., 2002). In line with their biological importance, the Era proteins are highly conserved at the level of sequence and structure even between most distant clades (Figures 2, 3). The integrity of both domains is strictly necessary for the Era function.

To date, 10 structures of Era proteins in various functional states have been solved. They broadly cover phylogenetically distant bacterial clades (*E. coli*, *Thermus thermophilus*, *Aquifex aeolicus*) and, more recently, capture human ERAL1 on a nascent mitochondrial SSU (Chen et al., 1999b; Tu et al., 2009; Tu et al., 2011; Harper et al., 2023). We refer the reader to the very complete review by Xinhua Ji which describes in detail the structural organization, the binding modes, and the switching behavior of bacterial Era (Ji, 2016). Here, we will just briefly discuss some salient features of the two Era domains and their interplay in the light of the existing structural, biochemical, and genetic data, with new insights brought about by recent mitochondrial ribosome biogenesis studies.

### The GTPase domain of Era

Like other TRAFAC GTPases, Era has a ∼170 aa-long G-domain with a characteristic fold in which a 6-stranded β-sheet is surrounded by 5 α-helices in a highly conserved alternating pattern that brings together five diagnostic motifs involved in GTP binding and hydrolysis (Figure 2). All β-strands are parallel, except for β2, which is uniquely antiparallel in all TRAFAC GTPases (Leipe et al., 2002). The *G1 motif* (*Walker A*) connects the strand β1 and the helix α1 and has a consensus sequence GxxxxGK(S/T). It is involved in the binding of α- and β-phosphates of GTP/GDP and for this reason often referred to as ‘*P-loop*’ (P for ‘phosphate’; hence the name of the entire superfamily possessing this motif—‘P-loop NTPases’). The *G2 motif,* in the loop between the helix α1 and the strand β2, has a consensus sequence TTR containing an invariant Thr residue that binds a Mg^2+^ ion required for GTP hydrolysis (Wu et al., 1995). The *G3 motif* (*Walker B*) is found right after the strand β3 and follows the consensus DTPG; it participates in Mg^2+^ and γ-phosphate binding. Finally, the *G4* and *G5 motifs,* located immediately after the strands β5 and β6 and having consensus sequences NKxD and SA, respectively, specifically recognize the guanine base of GTP/GDP (Bourne et al., 1991; Paduch et al., 2001).

Such an architecture of the active center explains why all Era proteins bind GTP and GDP with high affinity and specificity (Ahnn et al., 1986; Chen et al., 1999a). Interestingly, while dGTP can be bound quite tightly (i.e., the 2′-OH group of ribose is not important for the interaction), GMP or cGMP fail to associate with Era, likely because they form too few contacts with the G-motifs (Inada et al., 1989; Chen et al., 1990; Sullivan et al., 2000). ATP, UTP and CTP are not bound either since they are discriminated against by the G4 and the G5 motifs (Wu et al., 1995; Kawabata et al., 1997; Morimoto et al., 2002). Several studies showed that GDP binds to Era competitively and significantly more tightly than GTP. However, since both *K*_*d*_s are in the low micromolar range (i.e., much less than the intracellular concentrations of GDP and GTP), Era most probably cannot operate as a GDP/GTP sensor, as sometimes speculated (Chen et al., 1990; Bourne et al., 1991; Wu et al., 1995; Sullivan et al., 2000). Instead, similar to other GTPases, it works as a molecular switch, and this property appears to be essential for its cellular function.

### GTP hydrolysis and conformation switching

GTPases have become a paradigmatic example of how an enzymatically catalyzed chemical reaction can be coupled to mechanical movement. The resulting switching behavior, whereby the enzyme cycles between two different conformations, offers a simple and efficient mechanism driving forward a wide variety of biological processes, from translation to signal transduction (Bourne et al., 1991; Paduch et al., 2001). It is also a sensible way to rhythm the ribosome assembly. Indeed, several TRAFAC GTPases have been leveraged by bacteria, chloroplasts, and mitochondria to guide the biogenesis of both ribosomal subunits at specific assembly steps (Britton, 2009; Verstraeten et al., 2011; Goto et al., 2013; Maiti et al., 2021).

Structural studies by X-ray crystallography shed light on how Era uses GTP hydrolysis to change its shape (Tu et al., 2009). Free Era assumes one of two alternative conformations (Figure 3A). When it is bound to GTP (or its nonhydrolyzable analogue), all the G-motifs are involved in the interaction (Figures 2A,B), and the GTPase domain is rigid and closed. This is the ON-state of Era. However, upon GTP hydrolysis, the G2 and G3 motifs lose their ligands (Mg^2+^ and γ-phosphate), and the surrounding structural elements (so-called ‘*switches I and II*’) swing open. The resulting conformation is much looser and involves a significant movement of the adjacent loops and the helix α2. The apo-form of Era has essentially the same conformation (Chen et al., 1999b), i.e., both the apo- and the GDP-bound enzymes are in an inactive, OFF-state.

To make the cycling between the two conformations possible, a GTPase must be able to i) hydrolyze GTP to switch to the OFF-state and ii) exchange GDP for GTP to reset to the ON-state again (Figure 3A). GTP hydrolysis by Era occurs in a substrate-assisted manner: the γ-phosphate of GTP itself (activated by Mg^2+^) acts as a general base abstracting a proton from water; the resulting hydroxyl performs the nucleophilic attack on the γ-phosphate, with GDP fulfilling the role of the leaving group (Paduch et al., 2001; Tu et al., 2009). Similar to most switching GTPases, Era has poor intrinsic GTPase activity (Chen et al., 1990; Bourne et al., 1991; Wu et al., 1995; Chen et al., 1999a; Paduch et al., 2001). In fact, Era lacks an important Gln residue in the switch II that is responsible for aligning water for the nucleophilic attack in other small GTPases (such “incomplete” enzymes are called HAS-GTPases, for ‘*hydrophobic amino acid substituted*’—Mishra et al., 2005). Moreover, like all switching GTPases, Era also lacks a critical Arg residue required for the stabilization of the transition state. This residue is typically supplied in-*trans* by a GTPase activating protein (GAP) which, in the case of Era, remains unknown (Bourne et al., 1991; Paduch et al., 2001). However, like in many other TRAFAC GTPases, this role seems to be taken on by a potassium ion coordinated by two invariant Asn residues in the G1 motif and the so-called ‘*K-loop*’ imbedded in the switch I (Figure 2C). Addition of K^+^ (or similarly sized monocations) stimulates the GTPase activity of Era by an order of magnitude (Rafay et al., 2012; Shalaeva et al., 2018).

Switching GTPases also normally require a guanosine nucleotide-exchanging factor (GEF) to replace GDP with GTP and reset the GTPase to the ON-state (Bourne et al., 1991; Paduch et al., 2001). Strikingly, some biophysical data suggest that regardless its high affinity for both GDP and GTP, Era easily and rapidly exchanges them in solution. Both association and dissociation rates are so high that GDP can be replaced by GTP within seconds without the need of helper proteins. Therefore, the normally higher concentration of GTP in the cellular milieu must be sufficient a driver to enable unassisted resetting of Era into the ON-state (Sullivan et al., 2000). (See, however, the section “ERAL1 homeostasis in mammalian mitochondria” for a possible counterexample.)

The GTPase activity of Era is strictly required for its cellular function. Even apparently conservative changes in the G1 and G2 motifs or the switches (e.g., the K21R mutation in the G1 motif of *E. coli* Era) may not be tolerated and result in a lethal phenotype (Pillutla et al., 1995; Shimamoto and Inouye, 1996; Johnstone et al., 1999). Some milder mutations, that do not fully disrupt GTPase activity, produce viable but still severe phenotypes, such as heat and cold sensitivity, cell filamentation, significant growth delay, and inability to use certain carbon sources (Lerner et al., 1992; Lerner et al., 1995; Pillutla et al., 1996; Britton et al., 1997; Britton et al., 1998; Zhou et al., 2020). The N236I mutation in the G4-motif of human ERAL1 causes the Perrault syndrome (sensorineural deafness and ovarian dysgenesis) in humans (Chatzispyrou et al., 2017).

### The KH domain and RNA-binding activity of Era

Downstream of the GTPase domain, all Era proteins obligatorily possess an extended KH domain (Figure 2). This rather compact entity of ∼120 aa includes a core region formed by two parallel β-strands (βB and βC) separated by the helix-turn-helix motif (αB and αC), characteristic of all KH domains. Two additional elements—the N-terminally situated helix αA and the antiparallel strand βA—permit to classify this KH domain as *type II,* which is common in bacteria and bacteria-derived organelles. Finally, the extra helix αD extends the canonical KH fold on the C-terminal side, which is characteristic of the Era family (Chen et al., 1999b; Johnstone et al., 1999; Nicastro et al., 2015).

KH domains are classic RNA-binding elements present in a wide variety of proteins, often in multiple copies. They typically recognize 4 consecutive nucleotides of single-stranded RNA using a characteristic GxxG motif within the helix-turn-helix (Figure 2). The specificity of recognition differs between proteins and is conferred by multiple residues of the core KH-fold (Nicastro et al., 2015). In Era, the KH domain is responsible for its general RNA-binding ability and the specific association with the 3′-minor domain of the SSU rRNA, more precisely, h45 and the downstream single-stranded tail at the 3′-end of the molecule (Johnstone et al., 1999; Meier et al., 2000; Akiyama et al., 2001; Hang et al., 2001; Gohda et al., 2003; Hang and Zhao, 2003; Sun et al., 2019). This interaction has been studied in detail in bacteria by X-ray crystallography (Tu et al., 2009; Tu et al., 2011). Era was found to interact with an extended single-stranded region right after h45. Using the GxxG motif, it recognizes the highly conserved GAUCA sequence (especially, the two adenines), while the helix αC, the strand βC and the variable loop between βA and βB additionally enable binding of the downstream anti-Shine-Dalgarno sequence CCUCC. Finally, the top side of the domain forms multiple contacts with the base of h45 and the universally conserved guanine of the GAUCA sequence (Figures 2A,B). The helix h45 and the adjacent GACGA site seem to be particularly important for human ERAL1, since the mammalian mitochondrial SSU rRNA lacks the anti-Shine-Dalgarno sequence (Dennerlein et al., 2010; Harper et al., 2023).

Like the GTPase domain, the KH domain is required for the cellular function of Era. Its removal or mutations in the helix-turn-helix motif or in the h45-interacting loops on the top of the domain show a severe loss-of-function phenotype in various species (Pillutla et al., 1995; Zuber et al., 1997; Johnstone et al., 1999; Zhao et al., 1999; Hang et al., 2001; Gohda et al., 2003; Auvray et al., 2007; Tu et al., 2011; Voshol et al., 2015; Sun et al., 2019).

### Communication between the Era domains

The combination of a GTPase and an RNA-binding modules in one polypeptide begs the question whether Era may somehow coordinate their activities to time its intervention in the ribosome biogenesis pathway. Existing experimental evidence still struggles to provide a clear molecular mechanism for such a coordination. In this section, we will discuss disparate observations for and against the existence of a biologically relevant crosstalk between the two Era domains.

#### GTP binding regulates the interaction with the SSU rRNA

The idea that the conformational state of the GTPase domain, which depends on the ligand in the active center (Figure 3A), may influence the RNA-binding activity of the KH domain comes from X-ray crystallographic studies of bacterial Era complexes (Chen et al., 1999b; Tu et al., 2009; Tu et al., 2011). It is based on the striking observation that the apo- and the GDP-bound Era proteins demonstrate a rotated conformation of the KH domain, in which the negatively charged helix αD partially blocks access to the RNA-binding groove. By contrast, in the GTP-bound state, the KH domain is reoriented in such a way that RNA can access the binding site without problem (Tu et al., 2009). These findings suggested a tempting scenario where apo- or GDP-bound Era (OFF-state) is first re-loaded with GTP to switch to the ON-state, which licenses its interaction with the 3′-minor domain of the SSU rRNA (Figure 3A).

**FIGURE 4:**
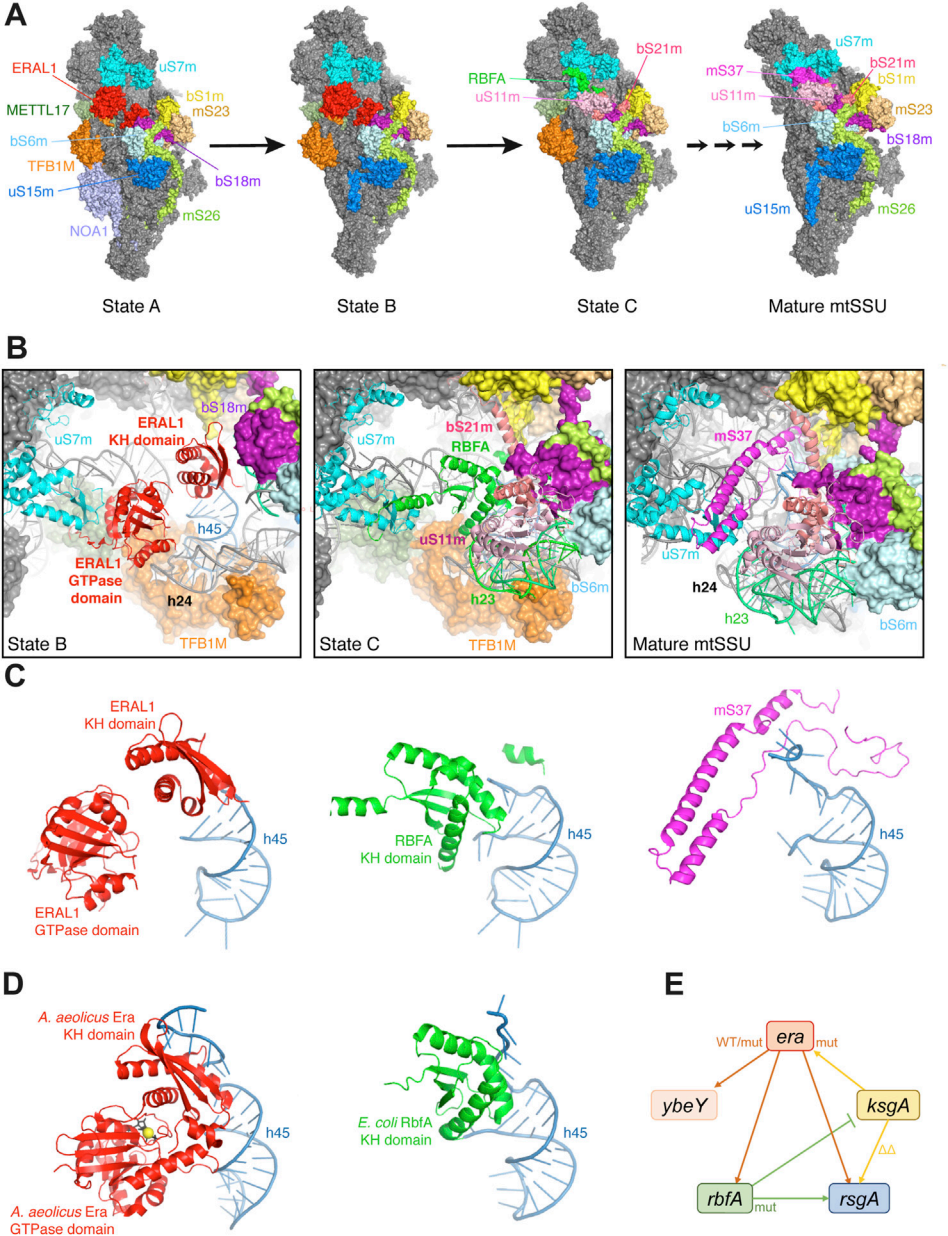
Place of Era in the ribosome biogenesis. **(A)** Cryo-EM structures of METTL17-containing human mitochondrial SSU assembly intermediates and the mature SSU (8csp, 8csq, 8csr, 7po3) (Itoh et al., 2022; Harper et al., 2023). All particles are shown from the platform side, and r-proteins and AFs surrounding the ERAL1-binding site are highlighted in different colors. **(B)** Zoom-in views of the same structures aligned relative uS7m, showing sequential changes in the occupancy by AFs, rRNA elements and r-proteins, and a progressive closure of the platform. The r-protein bS1m is yellow. **(C)** Comparison of the three mutually exclusive proteins (ERAL1, RBFA, mS37) sequentially interacting with h45 in the same structures. Note how the single-stranded 3′-tail of 12S rRNA gets progressively stabilized during assembly. **(D)** The homologous structures of a bacterial Era-GDPNP-h45 complex (3r9x) and an RbfA-containing SSU assembly intermediate (7bog) shown in the same orientation relative h45 as in **(C)** (Tu et al., 2011; Schedlbauer et al., 2021). **(E)** Genetic interactions between *era* and other genes involved in the platform maturation in *E. coli*. Overexpression of WT *era* suppresses the deletion of *rbfA*, *rsgA* and *ybeY* (Inoue et al., 2003; Campbell and Brown, 2008; Ghosal et al., 2018). The T99I mutation in the *era* gene also partially suppresses the *ybeY* deletion (Babu et al., 2022). By contrast, overproduction of the E200K Era mutant in the KH domain exacerbates the *rbfA* deletion (Lerner et al., 1995; Inoue et al., 2006)*,* while overexpression of *ksgA* in its turn suppresses the E200K *era* mutation (Lu and Inouye, 1998). For other interactions, see Campbell and Brown, 2008; Goto et al., 2011; Connolly and Culver, 2013; Naganathan and Culver, 2022.

However, this overall logical model is in an apparent disagreement with some *in vitro* observations showing that only apo-Era significantly binds to 16S rRNA and the mature SSU, whereas the addition of GDP or GTP abolishes these interactions (Sayed et al., 1999; Sharma et al., 2005). Furthermore, the recently resolved early mitochondrial SSU assembly intermediates (Figure 3A, inset) seem to contain ERAL1 in a nucleotide-free form, even though its conformation resembles the activated ON-state (Harper et al., 2023). This paradoxical property is unique among GTPase AFs, for which the apo- or GDP-associated form is systematically a poorer ribosome binder than the GTP-loaded one (Goto et al., 2013). It may reflect the existence of a more intricate, multi-state control of the Era-ribosome association by nucleotides. It is possible that Era is naturally recruited to the nascent SSU in the apo-state and acquires GTP later during assembly. Indeed, in the already mentioned mitochondrial SSU intermediates, the GTP-binding site of ERAL1 is fully exposed (see Figure 4B below), offering unhampered access for nucleotides (Harper et al., 2023).

#### SSU rRNA binding stimulates the GTPase activity

Deletion of the KH domain has been shown to slightly increase the affinity of *S. pneumoniae* Era for GTP, while significantly decreasing its GTPase activity (Zhao et al., 1999; Hang and Zhao, 2003). This suggests that the KH domain by itself influences the function of the N-terminal domain. Furthermore, multiple biochemical studies showed that the presence of RNA (especially 16S rRNA) or the SSU has a considerable stimulatory effect on the GTPase activity of bacterial Era (Meier et al., 1999; Meier et al., 2000; Loh et al., 2007; Corrigan et al., 2016). The helix h45 with the downstream single-stranded tail is sufficient to increase the rate of GTP hydrolysis by an order of magnitude, without affecting the *K*_*M*_ of the enzyme for GTP. Mutations of nucleotides involved in the interaction with the GxxG motif, especially the simultaneous replacement of both conserved adenines of the GAUCA sequence, abolish this stimulatory effect (Hang et al., 2001; Hang and Zhao, 2003; Tu et al., 2009; Tu et al., 2011).

However, how 16S rRNA stimulates GTP hydrolysis is unclear. Unlike GAPs, it binds very far from the GTPase active center and does not induce appreciable conformational changes (Tu et al., 2009; Tu et al., 2011). Furthermore, even though X-ray crystallography did identify several contacts between the two domains, suggesting at least some physical means to relay a mechanical movement between them, these data should be interpreted with caution. Crystal packing likely affected the native conformation of the protein, forcing non-physiological interactions normally absent in solution, such as Era dimerization via the switch II region and the anomalous sequestration of the switch I by the β-sheet of the KH domain observed in the apo- and GDP-bound crystal structures (Chen et al., 1999a; Chen et al., 1999b; Tu et al., 2009). It must be emphasized that in two cryo-EM structures of human mitochondrial SSU assembly intermediates, the two domains of ERAL1 are splayed apart (Figures 4A–C). Such an isolated position of the two domains makes a direct coupling of RNA binding and GTP hydrolysis more difficult (Harper et al., 2023). Moreover, if the binding of Era·GTP to the SSU rRNA immediately triggered GTP hydrolysis and Era release, it would hardly have time to perform its role as an assembly factor. Yet, Era is reputed to remain bound to the nascent SSU for a prolonged period of time, accompanying its early and intermediate assembly stages (Maksimova et al., 2022). As will be discussed in the section “Era in the ribosome assembly”, it is more likely that GTP hydrolysis and Era ejection are stimulated in a more complex way by a cooperative architectural rearrangement involving h45, other helices, r-proteins, and AFs.

#### Communication between the Era domains is functionally important

Even though we do not fully understand the mechanism by which GTP binding and hydrolysis govern Era recruitment and release, genetic evidence suggests that the coordination between the Era domains is very important. The simple ability to interact with the SSU is not sufficient. Overexpression of the KH domain alone, without the GTPase domain, was found to be toxic in various bacteria, provoking a strong ribosome biogenesis phenotype (Hang and Zhao, 2003; Inoue et al., 2006). Similarly, overexpression in human cells of *ERAL1* variants mutated in the G1 motif (and therefore incapable of GTP binding and hydrolysis) induced apoptosis, which could be fully suppressed by a simultaneous deletion of the KH domain (Akiyama et al., 2001). These observations suggest that too tight, uncontrolled binding of Era to h45, without an efficient ejection mechanism supplied by the GTPase domain, is counterproductive and may jeopardize ribosome biogenesis, leading to dire consequences.

Additional evidence for the importance of the inter-domain communication within Era comes from the analysis of genetic interactions with fellow AFs involved in the SSU platform biogenesis, such as RbfA. The overexpression of WT *era* suppresses the cold-sensitivity and other phenotypes associated with impaired ribosome assembly in Δ*rbfA E. coli* strains. However, if the linker between the two otherwise intact Era domains was extended by 8 amino acids, such a mutant could not suppress the *rbfA* deletion anymore, suggesting that a precise structural coordination between the Era domains is functionally critical (Inoue et al., 2003). Of note, the length of the linker is highly conserved from bacteria to humans (Figure 2C). Therefore, it is conceivable that Era, bound to the SSU in an extended conformation, could use the strain of the linker to relay h45 movements to the GTPase domain and *vice versa*, thereby coordinating GTP hydrolysis with the strength of its binding (Figure 4B).

### Era proteins with additional domains

Fusions of Era with other domains are extremely rare (Figure 3B), which is probably explained by significant steric constraints imposed by the nascent SSU, the molecular environment where Era normally functions (see the next section). By far the most frequent fusion type features YbeY attached N-terminally to Era, further emphasizing a privileged relationship between these two ribosome biogenesis factors which likely collaborate during the platform assembly (Liao et al., 2021). This architecture is found in several members of the Clostridia group, some Selenomonadales, and *Thermoleophilum album*. Fusions with RNase III are observed in some *Rickettsia* species. Other recurrently found domain combinations (e.g., with CS and SGS domains in Peronosporales and with DUF916 in certain *Streptomyces sp*.) are currently difficult to interpret due to scarce data about their molecular functions.

## Era in the ribosome assembly

Era specifically and strongly interacts with the SSU in bacteria, mitochondria, and chloroplasts (Sayed et al., 1999; Sharma et al., 2005; Inoue et al., 2006; Dennerlein et al., 2010; Uchiumi et al., 2010; Reyes et al., 2020; Méteignier et al., 2021; Valach et al., 2023). This said, one should clearly distinguish two kinds of Era-SSU associations described in literature: i) interactions with SSU assembly intermediates and ii) binding to the mature SSU. While these two phenomena are superficially similar and even share some structural principles, their biological significance is not the same (Sharma et al., 2005; Razi et al., 2019; Harper et al., 2023). Nevertheless, both scenarios provide important information about the role of Era in the ribosome biogenesis and help rationalize certain functional interdependencies between Era, other AFs, and r-proteins.

### Impact of Era on the SSU assembly

First indication that Era is required for the SSU assembly comes from phenotypic analyses of Era-deficient bacteria. In *E. coli* and *S. aureus*, mutations, knockdown or knockout of *era* result in the depletion of 70S ribosomes with a concomitant accumulation of individual subunits, suggesting that the SSU and the LSU cannot assemble into a monosome. As a result, the cellular protein synthesis slows down, severely compromising growth and survival (Nashimoto et al., 1985; Inoue et al., 2003; Loh et al., 2007; Razi et al., 2019; Wood et al., 2019; Bennison et al., 2021).

As expected from the binding specificity of Era, the defect is on the SSU side. Upon Era depletion, 16S rRNA shows a gross processing defect, which is a typical phenotype of an impaired SSU assembly also observed upon deletion of other SSU biogenesis factors, such as RimM, RbfA, RsgA, and YbeY (Nashimoto et al., 1985; Bylund et al., 1998; Inoue et al., 2003; Himeno et al., 2004; Davies et al., 2010; Baumgardt et al., 2018; Trinquier et al., 2019; Wood et al., 2019). Indeed, a recent cryo-EM analysis of ribosomes from Era-depleted *E. coli* revealed the accumulation of severely under-assembled SSU particles. Many of them had immature body and failed to assemble the platform and the head regions altogether. Some other particles continued their biogenesis past this stage by taking parallel assembly routes and mostly managed to shape the head and the body. However, they still could not stably recruit the r-proteins bS1, bS21, and uS11 to the platform and showed only partial association and fragmented densities for some head constituents (uS7, uS9, uS13, uS19). Furthermore, the helices h23, h24, h44 and h45, forming the platform and the 3′-minor domain regions, remained largely immature (Razi et al., 2019). Therefore, the absence of Era dramatically perturbed the assembly, which strongly destabilized and functionally impaired the SSU.

Similar results were obtained for eukaryotic Era proteins. In human cells, both *ERAL1* knockdown and the Perrault syndrome N236I mutation significantly destabilized the mitochondrial SSU, impacting the mitochondrial protein synthesis and respiration (Dennerlein et al., 2010; Uchiumi et al., 2010; Chatzispyrou et al., 2017). In rice, insertion into the *WSL6* locus, encoding a chloroplast Era homologue, resulted in the disappearance of chloroplast ribosomes and the disruption of the organellar translation (Sun et al., 2019).

### Placing Era into the SSU biogenesis pathway

The profound impact of Era on the SSU assembly notwithstanding (Tamaru et al., 2018), its exact molecular function remains elusive. However, the sum of biochemical, genetic, and structural evidence now enables us to formulate better-framed hypotheses as to where and when it acts. The flagrant deficiency of the platform assembly and the interaction with h45 strongly implicate Era in the maturation of the central and 3′-minor domains (Tu et al., 2011; Razi et al., 2019). Indeed, when mixed with mature ribosomes, Era interacts in the cleft between the head and the platform of the SSU (Sharma et al., 2005; Razi et al., 2019), and a similar binding pattern has been observed in native mitochondrial SSU assembly intermediates (Harper et al., 2023). These latter structures not only confirmed the place of Era intervention but also hinted at the timing of its recruitment, helping us reconstitute the possible order of molecular events with respect to other factors implicated in the platform maturation in bacterial-type ribosomes. We will describe them in more detail in the following section.

#### Era in the mitochondrial ribosome assembly

The assembly intermediates in question were captured via METTL17, a mitochondria-specific methyltransferase-like AF required for the head maturation during earlier stages of the SSU biogenesis (Shi et al., 2019; Harper et al., 2023). The two least mature particles, designated ‘*State A*’ and ‘*State B*’, contain mitochondrial Era (ERAL1) bound between the head and the still immature platform (Figure 4A). The two states only differ by the presence of NOA1 (YqeH in bacteria), a GTPase AF bound to the SSU body and preventing the premature docking of h44 and the r-protein mS38 (He et al., 2012; Harper et al., 2023). Otherwise, at the level of the ERAL1-binding site, these two states are equivalent. ERAL1, apparently in an ‘ON-state’ (Figure 3A), is stretched between the r-protein uS7m in the head and h45 in the platform, with which it interacts via its GTPase and KH domains, respectively (Figure 4B). The helices h45 and h24 are already close to their mature positions; the single-stranded tail of 12S rRNA is not visible, suggesting a flappy conformation, while the stem of h45 is anchored by TFB1M, the mitochondrial homologue of the universally conserved adenine methyltransferase KsgA/RsmA/Dim1 (Connolly et al., 2008). The platform is still open and loose, since h23 and the associated r-proteins of the central domain are not yet on their places.

The situation changes as the assembly proceeds to the ‘*State C*’. ERAL1 is ejected and replaced by another KH domain factor, RBFA (Rozanska et al., 2017; Itoh et al., 2022; Harper et al., 2023; Zhou et al., 2023). Just like ERAL1, it interacts with the base of h45 and stabilizes the proximal part of the single-stranded tail of 12S rRNA (Figure 4C). Interestingly, both AFs use KH domains to interact with overlapping elements around h45, but RBFA binds in the opposite direction with respect to the KH domain of ERAL1. Nevertheless, their binding is sterically mutually exclusive, making the ERAL1-RBFA ‘*KH switch*’ inevitable (Figures 4A–C). The platform in the State C is dramatically transformed: h23 is now docked on h24 and stabilized by the newly recruited uS11m, which additionally permits the stable incorporation of bS21m. The platform is now mostly closed and rigid: the considerably smaller RBFA works as “glue” by bringing closer uS7m and h45 (Figure 4B). Subsequent maturation steps will close the platform even further by substituting the slenderer mS37 for RBFA, which will ultimately stabilize the 3′-end of 12S rRNA (Figures 4A–C) (Itoh et al., 2022; Harper et al., 2023).

Based on the comparison of the States B and C, we can plausibly propose that ERAL1 prevents premature platform closure and may be involved in two key architectural events—the docking of h23 and the recruitment of uS11m and bS21 (Figures 4A,B). This hypothesis is supported by the known physical association between ERAL1 and YBEY, an AF required for the stable installation of uS11m in human mitochondria (Summer et al., 2020; D’Souza et al., 2021), suggesting that both the factors collaborate to shape the platform (Liao et al., 2021). The existence of an early ERA/TFB1M/NOA1-containing SSU assembly intermediate, analogous to the State A and preceding the RBFA recruitment, has been recently proposed based on coimmunoprecipitation-MS experiments in the extremely divergent mitochondrial system of *Diplonema papillatum* (Valach et al., 2023).

#### Era in the bacterial ribosome assembly

Much of what has been learned about Era from these mitochondrial studies may be valid for the bacterial ribosome assembly too. Indeed, bacterial Era and RbfA interact with h45 similarly to their mitochondrial orthologues (Figures 4C,D), and even a ternary complex between *A. aeolicus* Era, h45 and KsgA has been structurally characterized (Tu et al., 2011). The involvement of bacterial Era in the SSU platform assembly is further supported by strong genetic interactions with *ksgA*, *rbfA*, and *rsgA* (Figure 4E), all encoding factors associated with or close to h45 and driving late platform maturation steps (Lu and Inouye, 1998; Inoue et al., 2003; Inoue et al., 2006; Campbell and Brown, 2008). The overexpression of *era* ameliorates the ribosomal phenotypes of Δ*rbfA* and Δ*rsgA* cells (Inoue et al., 2003; Campbell and Brown, 2008; Wood et al., 2019). Although interpretation of epistatic effects is not straightforward (Naganathan and Culver, 2022), they seem to suggest that Era intervenes upstream of RbfA and RsgA: overexpression may delay the departure of Era from SSU assembly intermediates, offering them more time to find an alternative assembly route and obviate the need for some downstream AFs (Mulder et al., 2010; Thurlow et al., 2016). Indeed, the Era-dE mutant lacking the switch I (this mutant does not bind GTP and therefore cannot be easily ejected from the SSU following GTP hydrolysis) turned out to be an even better suppressor of ribosome assembly defects in the Δ*rbfA* strain: it fully restored the formation of 70S ribosomes and 16S rRNA processing (Pillutla et al., 1996; Shimamoto and Inouye, 1996; Inoue et al., 2003).

Analysis of SSU structures from *E. coli* deleted for *rsgA* and/or *rbfA* shows that their assembly defects are much milder compared to those of Era-depleted cells, arguing for an earlier involvement of Era in the platform assembly (Jomaa et al., 2011; Yang et al., 2014; Maksimova et al., 2021). Furthermore, recent cryo-EM studies of late SSU assembly intermediates in *E. coli* clearly suggested the following order of recruitment for AFs: KsgA → RbfA → RsgA. Importantly, in all these intermediates the platform is already consolidated and resembles the State C of the mitochondrial SSU (except for the r-protein bS21, which is recruited late in bacteria), suggesting that Era has already performed its molecular act and left (Schedlbauer et al., 2021). These considerations also exclude a direct participation of Era in the final 3′-processing of 16S rRNA, which is a very late event in bacteria and eukaryotes alike (Shetty and Varshney, 2016; Klinge and Woolford, 2019).

Copious evidence associates Era with YbeY (Liao et al., 2021). We already pointed out that the two proteins are sometimes fused into a single polypeptide (Figure 3B), suggesting that they function simultaneously during the SSU assembly. Stand-alone Era and YbeY proteins physically associate with each other in various bacteria (Vercruysse et al., 2016; Wood et al., 2019). They also interact genetically: overexpression of WT *era* (Ghosal et al., 2018) and a spontaneous point mutation in its GTPase domain (Babu et al., 2022) were shown to significantly ameliorate the growth and the ribosomal phenotypes of Δ*ybeY E. coli*. Since the molecular function of YbeY is tightly connected with the ribosomal protein uS11, in bacteria as in mitochondria (Vercruysse et al., 2016; Summer et al., 2020; D’Souza et al., 2021), it might be that Era and YbeY cooperate in installing uS11, thereby enabling the platform closure, like it happens in mitochondria (Summer et al., 2020; Liao et al., 2021; Harper et al., 2023). Indeed, *in vitro E. coli* SSU reconstitution experiments showed that addition of Era significantly accelerated the recruitment of uS11 (Bunner et al., 2010). Furthermore, *in vivo* DMS footprinting of *E. coli* ribosomes showed that Era depletion significantly exposed h23 and h24, in agreement with the cryo-EM structures, whereas Era re-expression resulted in the compaction of these helices (Razi et al., 2019). Therefore, the proper positioning of uS11 and h23 may be part of a deeply conserved molecular mechanism used by Era to shape the platform in various bacterial-type ribosomes.

### A ribosome “unmaturation” factor?

First studies of Era binding to bacterial ribosomes were carried out *in vitro* with the use of a purified Era proteins and mature 30S subunits (Sayed et al., 1999; Sharma et al., 2005; Razi et al., 2019). These fully assembled SSUs were unlikely Era substrates as they are obviously very different from native SSU assembly intermediates (Mulder et al., 2010). Nevertheless, Era showed a strong affinity toward bS1-depleted 30S subunits (Sharma et al., 2005; Razi et al., 2019). Moreover, excess of Era prevented the reassociation of mature 30S and 50S subunits into 70S monosomes (Sharma et al., 2005). Finally, addition of a 5-fold excess of Era in the presence of GDP, GTP or GDPNP was sufficient to split 70S monosomes into individual subunits (Razi et al., 2019). These puzzling observations, quite unusual for an AF, begged several questions: i) how does the Era-SSU association occur? ii) what happens to mature ribosomes upon Era binding? iii) what is the biological significance of these phenomena?

An early cryo-EM study showed that upon coincubation of bS1-depleted 30S subunits from *T. thermophilus* with Era, a new bilobed density appeared in the cleft between the head and the platform (Sharma et al., 2005). Its position and overall shape are reminiscent of those of ERAL1 on mitochondrial SSU assembly intermediates (Harper et al., 2023), suggesting a similar binding mode. Era is wedged in such a way that the 50S subunit cannot join the 30S subunit anymore; it occupies the binding site of the r-protein bS1, which in bacteria is large, loosely bound and required for translation initiation (Sengupta et al., 2001). This coarse-grained structure rationalizes the 70S ribosome splitting and anti-association activities of Era *in vitro* and predicts that its binding to mature 30S subunits would interfere with translation. Unfortunately, the low resolution of this structure precludes reliable assignment of molecular interactions (Sharma et al., 2005).

More recently, an analogous experiment performed with *E. coli* Era and 30S subunits in the presence of GDPNP yielded a cryo-EM structure with a 3.9 Å resolution (Razi et al., 2019). Although the density between the head and the platform potentially corresponding to the bound Era was highly fragmented, making structural details of this interaction inaccessible, this structure turned out to be revealing with respect to the impact Era had on the mature 30S subunit. The head and the platform became more mobile; the helix h44, a key part of the decoding center, was virtually invisible, indicating its high flexibility. Additionally, the densities for tips of h23 and h24 were fragmented, and no densities could be observed for bS1, uS7, bS21, and the N-terminal part of uS13. Overall, the binding of Era appears to have ‘undone’ some of of the SSU assembly steps and critically destabilized its functionally essential regions, making it initiation-incompetent. In a way, Era reverted the SSU to a state more similar to its normal substrate encountered during the SSU biogenesis (Figures 4A,B).

The relevance of this striking phenomenon remains enigmatic. While a similar (albeit subtler) “unmaturation” activity was described for RbfA and RsgA, it required in all cases co-incubation of 30S subunits with an excess of the AF (Razi et al., 2017; 2019; Bikmullin et al., 2023). However, the *in vivo* abundance of Era in bacteria does not exceed ∼5% of that of ribosomes, making such conditions difficult to achieve inside the cell (Chen et al., 1990; Morimoto et al., 2002). The proposed hypotheses that Era may perform “unmaturation” of 30S subunits under particular stress conditions or help split hibernating ribosomes remain valid possibilities warranting experimental investigation (Razi et al., 2019).

## Era in cell and organism physiology

To appreciate how important Era really is for the cell, it is sufficient to have a look at the multiple phenotypes caused by its mutation or complete loss. Many of them, such as lethality, impaired protein synthesis and growth, and cold sensitivity, are obviously linked to its role in the ribosome assembly; they were discussed in previous sections. Others are more difficult to explain: they reflect the extreme pleiotropy of physiological manifestations usually associated with impaired central cellular machineries and often having complex, meandering etiologies. Without aspiring to decipher the mechanisms behind these intricate phenotypes, we will provide in this section a brief overview of the most striking features distinguishing Era-deficient organisms.

### Significant phenotypes of Era deficiency in bacteria

#### “Cell cycle regulator”

A series of early studies connected Era to bacterial replication and division and for decades coined it as a “cell cycle regulator”. Specifically, it was observed that, upon Era depletion, *E. coli* stops proliferating, and the bacteria get more and more elongated, until they lyse. These filamentous cells lack septa and show multiple properly segregated nucleoids, pointing at a division defect unrelated to DNA replication (Gollop and March 1991). Interestingly, while the bacteria stop dividing ∼2 h after the beginning of Era depletion, the filamentation occurs much later, suggesting that it is a secondary consequence of a more fundamental primary defect which may be related to the disruption of protein synthesis (Gollop and March 1991).

Similar septation phenotypes were also described for a few unviable *E. coli era* mutants (Johnstone et al., 1999) and even for the viable *era647* and P17R mutants affecting the GTPase domain (Britton et al., 1997; Britton et al., 1998; Zhou et al., 2020). A striking feature of the latter mutation (which on its own is quite deleterious) is that it suppressed a number of temperature-sensitive mutations in DNA replication (*dnaB*, *dnaG*) and chromosome partitioning (*gyrB*, *parC*, *mukB*) genes, while exacerbating those related to the septum formation (*ftsZ*, *ftsA*) (Britton et al., 1997; Britton et al., 1998). Vice versa, overexpression or hypermorphic alleles of *ftsZ* suppressed the filamentation of *era647*-expressing cells, suggesting that behind the *era*-associated cell division phenotype there may be a defect in FtsZ-mediated Z-ring formation (Zhou et al., 2020). However, it must be noted that not all *era* mutations result in cell division phenotypes: several cold-sensitive *E. coli* strains (N26S, A256D, E200K) did not show any filamentation when shifted to a non-permissive temperature, even though their growth was drastically inhibited (Lerner et al., 1995).

Superficially similar, but probably mechanistically different, phenotypes were described in *B. subtilis*. Era depletion to less than 10% of its normal level caused the cells elongate up to 2-fold, whereas complete deletion dramatically affected growth and viability, caused extensive cell filamentation, and resulted in diffuse nucleoids (Minkovsky et al., 2002; Morimoto et al., 2002). This defect was associated with abnormally increased replication initiation (Morimoto et al., 2002). In the cyanobacterium *S. elongatus*, an *era* mutant that had lost the last 20 aa of the KH domain due to a transposon insertion showed an increased number of highly elongated cells with anarchically positioned constrictions. Complete *era* deletion in this bacterium yielded filamentous cells that failed to thrive (Voshol et al., 2015).

The sum of these observations, along with the frequent synteny between the *era* and DNA primase-encoding *dnaG* genes (Figure 1B), betray the existence of a tight--but so-far mechanistically elusive--link between the function of Era and cell division across bacterial species. Nevertheless, it is still unclear to what extent the “cell cycle” phenotypes are specifically Era-related. The depletion of other ribosome assembly GTPases (ObgE, Der, EngB, RbgA) in *B. subtilis* resulted in even stronger cell elongation, while the removal of YqeH similarly led to over-initiation of chromosome replication (Morimoto et al., 2002). The LSU assembly factor ObgE has been even more tightly linked to DNA replication and segregation in multiple species (Britton, 2009; Verstraeten et al., 2011).

#### Era and cellular metabolism

Without surprise, *era* mutants are usually metabolically altered. For example, the overexpression of the Era-dE variant, lacking the entire switch I region, renders *E. coli* incapable of growing on pyruvate or TCA intermediates (citrate, α-ketoglutarate, succinate, fumarate, malate) as the sole carbon source. However, when grown on glucose, this mutant had 2-3-fold increased ATP levels, presumably due to decreased ATP utilization in energy-consuming processes, such as protein synthesis (Pillutla et al., 1996). On the other hand, Era depletion in *S. mutans* made the GDP level rise above that of GTP, indicating a major metabolic shift (Baev et al., 1999).

Another connection to central metabolism came from an analysis of genetic interactions in *E. coli*. Disruption of *ptsN*, encoding the IIA^Ntr^ component of the phosphoenolpyruvate:sugar phosphotransferase system, was sufficient to overcome the lethality of a thermosensitive *era* mutation. Another suppressor mutation truncated *rpoN*, encoding the σ^N^-factor and located in the same operon as *ptsN* (Powell et al., 1995). These associations are intriguing as they suggest a connection between Era and the carbon/nitrogen metabolism via the PTS^Ntr^ phosphorelay system, which is reciprocally regulated by glutamate and α-ketoglutarate (Lee et al., 2013). However, the mechanism underlying the suppression of the *era* mutation remains unclear, especially since Δ*ptsN* phenotypes are known to be strain-dependent (Reaves and Rabinowitz, 2011).

In Terrabacteria, Era also seems to be important for anabolic processes. For example, the *S. elongatus* KH domain-truncated *era* mutant described in the previous section had an overall increased lipid content and altered ratios between different lipid types, such as hydrocarbons and certain fatty acids (Voshol et al., 2015).

#### Stress phenotypes

Numerous studies reported that Era-deficient bacteria are sensitive to abiotic and biotic stresses. *era* mutant strains were found to overproduce chaperones DnaK and GroEL and the stress-induced metalloproteinase LoiP (Pillutla et al., 1996; Huang et al., 2007; Lütticke et al., 2012). Deletion of *loiP* negatively affected the growth of the P17R *era* mutant, whereas its overexpression was beneficial (Huang et al., 2007). These observations suggest that Era-deficient bacteria are constitutively stressed.

Era-depleted *S. mutans* bacteria were heat-sensitive and grew poorly at 45°C or under mildly acidic or high-osmolarity conditions (Sato et al., 1998; Baev et al., 1999). These general abiotic stress phenotypes predict poor performance in pathogenesis-relevant conditions. Indeed, a *S. pneumoniae* Δ*era* mutant was mildly attenuated in a murine respiratory tract infection model (Zalacain et al., 2003). A *L. monocytogenes era* mutant with a truncated KH domain showed poor adhesion to inert surfaces (Auvray et al., 2007).

### Era deficiency in eukaryotes

Although eukaryotic Era homologues are only required for the assembly of mitochondrial and chloroplast ribosomes, which represent but a minor proportion of all cellular ribosomes, the essential nature of organellar translation for most aerobic eukaryotes makes them absolutely indispensable. Beyond a direct effect on the protein synthesis in the organelles, which was the focus of the previous sections of this review, defects in eukaryotic Era have far-reaching physiological consequences at the cellular and organismal levels. Below, we will briefly discuss these “secondary” phenotypes on the example of animal and plant Era homologues.

#### Era in animal cell physiology: autophagy and apoptosis

Era deficiency in mitochondria is often equated with mitochondrial dysfunction, which may have a wide spectrum of manifestations. Depletion of ERAL1 in HeLa cells resulted in growth arrest and caspase-dependent apoptosis, especially when they were cultured in galactose-containing media (Uchiumi et al., 2010; Xie et al., 2012). When grown on glucose, *ERAL1* knockdown cells underwent apoptosis only if mitochondria retained their genome, whereas ρ_0_ cells were immune to this effect (Dennerlein et al., 2010). These results indicate that the apoptotic response to the ablation of *ERAL1* expression is caused by the disruption of mitochondrial gene expression. Besides a direct effect the inhibition of the mitochondrial protein synthesis had on respiration, it also decreased mitochondrial membrane potential and increased reactive oxygen species (ROS), which triggered autophagy through the TP53-DRMA1 pathway (Uchiumi et al., 2010; Xie et al., 2012). It was noted that autophagy preceded--and to some extent delayed--apoptosis in ERAL1-deficient cells (Xie et al., 2012). Analogous observations were made in chicken lymphoma B-cell line DT40, where depletion of ERA resulted in a cell cycle arrest, growth inhibition, and increased cell death 4 days post promotor repression. The apoptosis was partially suppressed by the overexpression of Bcl-xL and completely by the introduction of human ERAL1. However, an ERAL1 variant with a truncated KH domain, incapable of RNA binding, could not rescue the ERA-deficient cells (Gohda et al., 2003).

Interestingly, overexpression of some *ERAL1* G1 motif mutants in HeLa cells also induced apoptosis in a dominant-negative way, which could be suppressed by co-overexpression of Bcl-xL or Bcl-2. By contrast, the same mutants truncated in the KH domain were not apoptogenic anymore. This finding suggests that ERAL1 variants which retain affinity for mitochondrial SSUs but fail to bind and hydrolyze GTP ultimately trigger the intrinsic apoptotic pathway (Akiyama et al., 2001).

#### Era in animal and human physiology: fertility and the Perrault syndrome

Era deficiency in animals has a profound effect at the organismal level. A complete *ERAL1* knockout in mice is lethal at embryonic day 13.5, in agreement with its requirement for the mitochondrial translation (Li et al., 2021). The ∼50% knockdown of the *Caenorhabditis elegans ERAL1* orthologue *E02H1.2* did not affect development but decreased oxygen consumption and resulted in female infertility by completely blocking egg production (Chatzispyrou et al., 2017).

In humans, there is currently one report of a disease-associated *ERAL1* mutation (Chatzispyrou et al., 2017). Three unrelated patients diagnosed with the Perrault syndrome were found to have a homozygous c.707A>T (p.N236I) mutation in the G4 motif of the GTPase domain, that replaced an absolutely conserved Asn required for the recognition of the guanine base of GTP (Figure 2). The Perrault syndrome (MIM 233400) is a rare, autosomal recessive mitochondrial disease characterized by sensorineural deafness and ovarian dysgenesis, leading to amenorrhea and infertility in women (Perrault et al., 1951). Besides *ERAL1*, it can be caused by mutations in nuclear genes encoding other proteins involved in the mitochondrial translation, such as the catalytic subunit of the mitochondrial RNase P PRORP, the aminoacyl-tRNA synthetases HARS2 and LARS2, and the putative mitoribosome maturation factor RMND1 (Hochberg et al., 2021; Faridi et al., 2022). In the *ERAL1*-associated cases, the molecular pathology is transparent: an Era protein with such a mutation is almost certainly functionally impaired (see the section “Structure-function of Era at the molecular level”). Additionally, it was found to be heavily depleted in patient skin fibroblasts, likely due to lower *in vivo* stability, which must have further compounded its functional deficiency. The mitochondrial SSU was selectively depleted, without gross changes in mobility by blue-native gel electrophoresis. Mitochondrial translation and respiration were significantly decreased, indicating a typical OXPHOS deficiency (Chatzispyrou et al., 2017).

Strikingly, a recent report identified a patient with the Perrault syndrome who possessed a compound heterozygous mutation (c.373A>T/p.K125stop, c.536G>A/p.R179H) in the *MRPS7* gene (Kline et al., 2022). *MRPS7* encodes the uS7m protein that directly binds and anchors the GTPase domain of ERAL1 in the nascent mitochondrial SSU (Figures 4A,B). Another homozygous *MRPS7* mutation (c.550A>G, p.M184V), found in two patients, resulted in sensorineural deafness combined with lactic acidemia, progressive renal and hepatic failure and, at least in one of them, primary hypogonadism (Menezes et al., 2015); it was recently proposed to be reclassified as Perrault syndrome (Kline et al., 2022). This mutation destabilized uS7m and strongly decreased 12S rRNA levels, indicative of a SSU assembly defect, which resulted in an impaired mitochondrial translation and an OXPHOS deficiency (Menezes et al., 2015). These findings suggest that both the *ERAL1*- and the *MRPS7*-associated cases have closely related molecular mechanisms rooted in a severely perturbed mitoribosome biogenesis.

#### Era in animal physiology: antiviral response

More recently, ERAL1 was proposed to play an unexpected moonlighting role in innate antiviral immunity. During infection with RNA viruses, ERAL1 was found to be required for normal type I interferon production, which is probably unrelated to its primary role in sustaining mitochondrial translation. Mice with *ERAL1* haploinsufficiency were more susceptible to vesicular stomatitis virus infection. Mechanistic studies suggest that upon viral infection (or simple introduction of 5′-triphosphorylated dsRNA into cells), ERAL1 translocates from mitochondria to the cytosol via the permeability transition pore. In the cytosol, it interacts with MAVS and favors its polymerization, contributing to the RIG-I/MDA5-mediated antiviral signaling cascade. Interestingly, although the interaction with MAVS depends on the KH domain, the RNA-binding ability of ERAL1 does not seem to be involved in its signaling function: ERAL1 neither colocalized with viral dsRNA nor had an impact on its accumulation (Li et al., 2021).

#### Era in plant physiology

The importance of mitochondrial ERA (ERG, ERG2) has been studied in two flowering plant species, *Antirrhinum majus* and *Arabidopsis thaliana* (Ingram et al., 1998; Cheng et al., 2018). This protein is produced ubiquitously, with a particularly high expression in actively dividing and metabolically active tissues (inflorescence tips, ovules, leaf veins, mature pollen). In both cases, a homozygous *ERG2* disruption is embryonic lethal, and even heterozygotes have pronounced developmental phenotypes. In *A. majus*, a quarter of heterozygous seeds contained shriveled embryo sacs with an uncellularized endosperm and finally aborted (Ingram et al., 1998). In *A. thaliana*, a sporophytic maternal effect phenotype was preponderant: siliques were shortened 3 days after pollination, and most heterozygous seeds were arrested in development already 1.5 days after pollination, showing extensive tissue degradation inside the embryo sacs. These events were accompanied by impaired mitochondrial translation, increased ROS accumulation, and apoptosis, similar to *ERAL1* ablation in animal cells (Cheng et al., 2018).

Chloroplast Era (WSL6, ERA1) was implicated in the SSU assembly, as a partner of the mTERF9 protein, in *A. thaliana* (Méteignier et al., 2021) and phenotypically studied in rice, where several so-called ‘*white striped leaf*’ mutations were mapped to genes involved in the chloroplast ribosome biogenesis (Tan et al., 2014; Wang et al., 2017; Liu et al., 2018; Sun et al., 2019). This name refers to the characteristic appearance of young mutant plants: after the first green leaf, all subsequent leaves are white-striped, with reduced chlorophyll and carotenoid contents and abnormal, thylakoid-deficient chloroplasts. As in the case of bacterial Era deficiency, this phenotype was cold-sensitive: at 20°C, the *WSL6* plants became completely albinic. In agreement with the role Era plays in the SSU biogenesis in other genetic systems, the *WSL6* chloroplasts showed a severe disruption of translation: the plastid ribosomes were depleted, and the protein synthesis was largely abolished. Therefore, WSL6 is indispensable for the early development of chloroplasts (Sun et al., 2019).

## Regulation of Era

**FIGURE 5:**
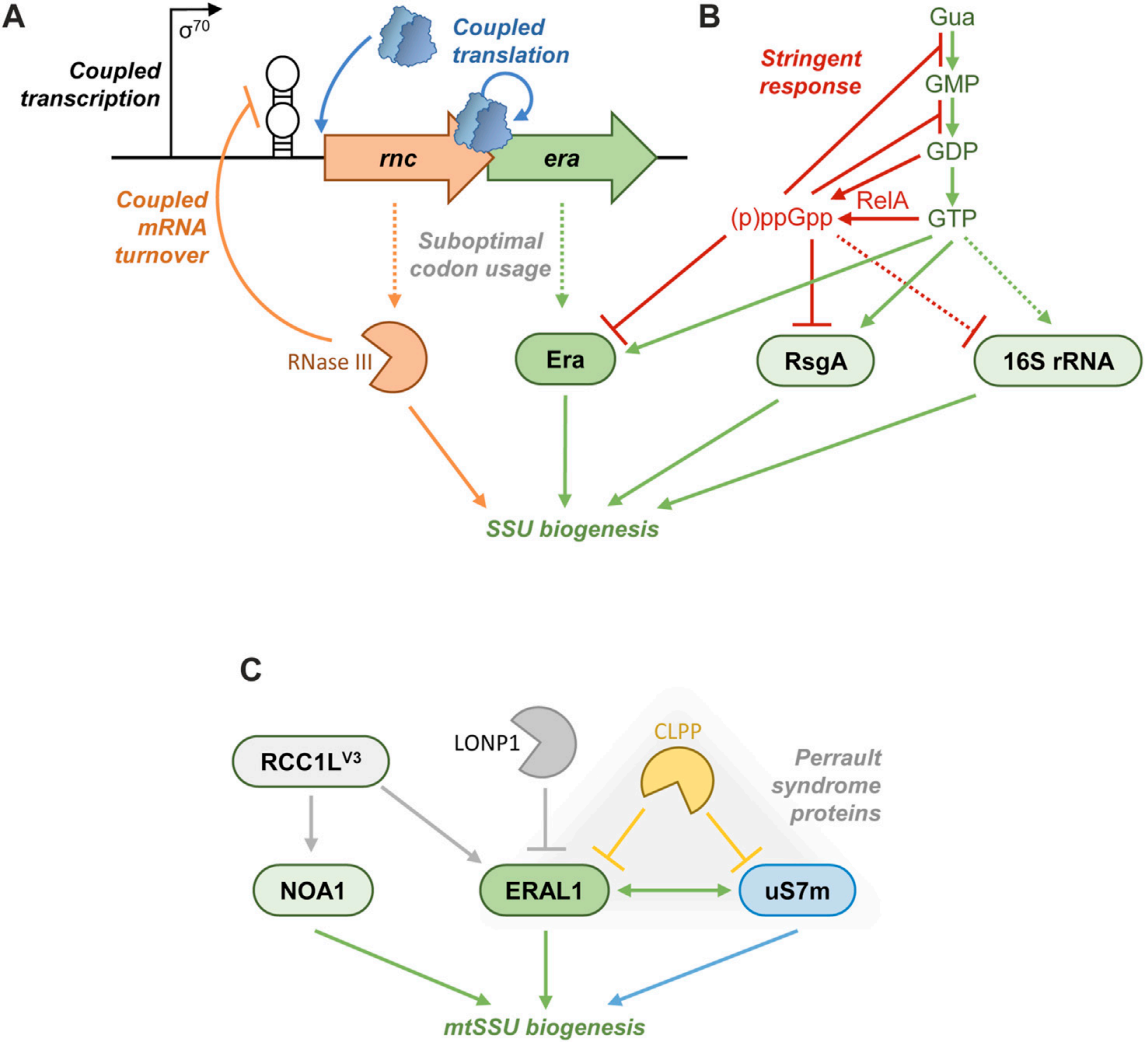
Regulation of Era. **(A)** In many Proteobacteria, Era is encoded downstream of RNase III, and their expression is tied at transcriptional (shared promoter), post-transcriptional (autoregulatory mRNA degradation by RNase III), and translational (re-initiating ribosomes) levels. Both proteins are produced at a relatively low abundance due to a poor codon usage. **(B)** In both *E. coli* and Terrabacteria, the stringent response exploits a variety of mechanisms to curb translation. During amino acid starvation, RelA consumes GTP and GDP and synthesizes large amounts of (p)ppGpp. The drop of GTP concentration curtails rRNA transcription in Terrabacteria, whereas in *E. coli* (p)ppGpp actively represses RNA polymerase at rRNA promoters. The perturbed balance between GTP and (p)ppGpp directly affects GTPases involved in the ribosome biogenesis and translation, as part of a rapid response to starvation. (p)ppGpp competitively overtakes such AFs as Era and RsgA, inhibiting their activity and stalling the SSU biogenesis. **(C)** ERAL1 control in mammalian mitochondria. RCC1L^V3^ is a putative GEF that positively regulates the association of ERAL1 and NOA1 with the mitochondrial SSU. By contrast, the proteases LONP1 and CLPP degrade ERAL1. The ERAL1-interacting r-protein uS7m is also a substrate of CLPP; these three proteins form a functional module associated with the Perrault syndrome.

As an assembly factor critical for the ribosome biogenesis, Era is perfectly positioned to control the bulk protein synthesis, and many organisms evolved strategies to regulate its production, activity, and turnover. In *E. coli*, the cellular level of Era follows the growth rate to match the ever-changing need in ribosomes (Britton et al., 1998). In *B. subtilis*, *era* additionally seems to be under control of the master regulator of sporulation Spo0A, which is responsible for the elevated production of Era in the postexponential phase. In line with this finding, *era*-deficient *B. subtilis* is dramatically impaired in viability and sporulation in stationary phase (Minkovsky et al., 2002). The latter example shows that the regulation of Era may sometimes take unexpected turns to serve specific needs of each particular species. Below, we will discuss some relatively well-studied regulatory mechanisms by which bacteria and mammals maintain the optimal level of Era activity to moderate their ribosome biogenesis (Figure 5).

### Post-transcriptional regulation in bacterial *rnc-era* operons

Many γ-proteobacteria tightly coordinate the expression of RNase III and Era, both of which are involved in early stages of the SSU biogenesis. This co-regulation occurs at transcriptional, post-transcriptional, and translational levels (Figure 5A). The two genes are co-transcribed from a shared σ^70^ promoter to yield a polycistronic mRNA (Ahnn et al., 1986; Bardwell et al., 1989; Takiff et al., 1989; Matsunaga et al., 1996a; Powell et al., 1999). They are also translationally coupled: the stop-codon of the *rnc* ORF, encoding RNase III, overlaps the start-codon of the *era* cistron. The translation of *era* is apparently contingent on that of *rnc* through a re-initiation mechanism, which creates a simple means of stoichiometric control between the two proteins (Ahnn et al., 1986; Chen et al., 1990; Anderson et al., 1996; Powell et al., 1999). As an additional mechanism of co-translational control, both the ORFs have a suboptimal codon usage in *E. coli*, congruent with their relatively low level of production (Takiff et al., 1989; Chen et al., 1990). On top of this, like many globally acting RNA-binding proteins (Smirnov, 2022), RNase III is subject to autoregulation to avoid potentially toxic overproduction. It cleaves a long stem-loop structure in the 5′-UTR of the *rnc-era* mRNA, provoking its degradation (Matsunaga et al., 1996a; Matsunaga et al., 1996b). By this means, the expression of *era* is concomitantly downregulated by RNase III (Bardwell et al., 1989; Anderson et al., 1996).

### Metabolic control of Era: stringent response

A direct control of GTPase activity of *E. coli* Era by small molecules has been proposed early on. Owing to its role in carbohydrate metabolism (see the section “Era and cellular metabolism”), multiple central metabolites had been tested *in vitro*, and acetate and 3-phosphoglycerate were found to strongly stimulate GTP hydrolysis, whereas glyceraldehyde 3-phosphate acted as an inhibitor (Meier et al., 2000). The *in vivo* relevance of these biochemical observations is still unknown.

Much better studied and likely more physiologically important is the connection between Era and the alarmones (p)ppGpp at the heart of the stringent response (Figure 5B). pppGpp and ppGpp are synthesized in an ATP-dependent manner from GTP and GDP, respectively, by RelA-like enzymes broadly conserved in bacteria and plant chloroplasts. The activity of RelA is turned on in a tRNA/ribosome-dependent manner upon amino acid starvation and results in a rapid accumulation of both alarmones in the cell. Additional stresses (osmotic shock, starvation for fatty acids, phosphate, or iron) trigger (p)ppGpp production by a related enzyme, SpoT, which also has the ability to degrade the alarmones (Potrykus and Cashel, 2008; Irving et al., 2021). In *E. coli*, (p)ppGpp, eventually helped by the DksA protein, binds to RNA polymerase and differentially affects its activity on cellular promoters. Namely, (p)ppGpp fully represses the rRNA transcription to prevent the production of new ribosomes. By contrast, it activates amino acid operons to replenish their stocks (Paul et al., 2004; Paul et al., 2005). In Terrabacteria, the mechanism is slightly different: (p)ppGpp does not bind to RNAP, and the shear depletion of the cellular GTP pool caused by the alarmone synthesis directly abolishes the rRNA transcription (Krásný and Gourse, 2004; Kasai et al., 2006). All these measures allow the bacteria to optimize their resource management during starvation, avoid further stress, restore metabolic equilibrium, and ultimately resume growth (Potrykus and Cashel, 2008; Irving et al., 2021).

The stringent response has another important dimension: (p)ppGpp can interact with guanosine factor-dependent or -related proteins and directly modulate their activity. In diverse bacteria, the alarmones competitively inhibit enzymes of the GTP biosynthesis and salvage pathways (Kriel et al., 2012; Liu et al., 2015; Corrigan et al., 2016; Zhang et al., 2018; Irving et al., 2021). By contrast, (p)ppGpp potently stimulates its own producer RelA (Shyp et al., 2012). Both these events create a positive feedback mechanism switching the cell to the stringent response mode with a completely remodeled gene expression (Irving et al., 2021). Ribosome-associated GTPases were on the list as just too obvious targets, and several laboratories gathered compelling evidence that the stringent response--and (p)ppGpp in particular--directly impact on the ribosome biogenesis by controlling GTP-dependent AFs in various bacterial species. These results were recently comprehensively reviewed (Zegarra et al., 2023). Here, we will primarily focus on the relationship between Era and the alarmones.

Naturally affine for G-nucleotides with at least two phosphates, Era from *E. coli* and *S. aureus* binds (p)ppGpp with affinities close to those for GDP and GTP and using the same binding site (Corrigan et al., 2016; Zhang et al., 2018). Since the 3′-pyrophosphate moiety is not specifically recognized by other crystallized TRAFAC GTPases (Buglino et al., 2002; Pausch et al., 2018), the association constant must be largely determined by the status of the 5′-pyrophosphate. Thus, ppGpp and GDP bind Era similarly well (in the low-micromolar range) and significantly tighter than GTP and pppGpp (Corrigan et al., 2016; Zhang et al., 2018; Bennison et al., 2021; Zegarra et al., 2023). However, under optimal conditions, GTP is much more abundant in the cell than GDP and (p)ppGpp, meaning that Era is found primarily in a GTP-bound state.

However, during the stringent response, RelA generates up to 1–4 mM (p)ppGpp, which is now on the same foot as GTP, if not higher (Cashel, 1975; Varik et al., 2017). This creates conditions where the pool of Era (and other ribosome assembly GTPases, such as RsgA, RbgA, Der, ObgE) massively shifts to a (p)ppGpp-bound state (Corrigan et al., 2016; Bennison et al., 2021; Zegarra et al., 2023). This state appears to be unproductive: (p)ppGpp inhibits their GTPase activity (Corrigan et al., 2016; Pausch et al., 2018). Judging by the available structures of RsgA, RbgA and ObgE in complex with alarmones, (p)ppGpp blocks these AFs in an inactive conformation, likely because the 3′-pyrophosphate sterically interferes with the recruitment of the G2 motif (Buglino et al., 2002; Pausch et al., 2018; Bennison et al., 2021). Since the organization of the active center is very similar between ribosome-associated GTPases (Leipe et al., 2002; Goto et al., 2013), these findings suggests that (p)ppGpp-bound Era cannot operate its normal switching cycle during the SSU assembly either. Furthermore, (p)ppGpp considerably weakened the ribosome-binding ability of Era (Bennison et al., 2021). All these factors imply that upon stringent response, the ribosome assembly rapidly (within ∼2 min) stalls in part because Era and other GTPases cannot function anymore. The arrest of the SSU biogenesis is especially obvious, as it results in the accumulation of particles lacking the r-proteins uS2, uS3, uS14 and bS21 and containing unprocessed 16S rRNA (Trinquier et al., 2019; Wood et al., 2019; Zegarra et al., 2023). This immediate response, mediated by GTP depletion and a direct inhibitory action of the alarmones, permits the cell to halt the ribosome production much faster than the indirect mechanism involving transcriptional rRNA repression (Potrykus and Cashel, 2008; Irving et al., 2021; Zegarra et al., 2023).

Although this aspect has not been well studied, the (p)ppGpp binding by Era (and, consequently, its activity) might be regulated by associated proteins. In *S. aureus*, Era directly interacts with Rel_Sau_, one of three (p)ppGpp synthases/hydrolases in this bacterium. Rel_Sau_ was found to mildly stimulate the GTPase activity of Era (Wood et al., 2019). In *E. coli*, SpoT interacts with the key partner of Era, YbeY, suggesting that all three proteins might be closely associated (Vercruysse et al., 2016). Interestingly, one study reported a direct interaction between Era and the nonspecific nucleotide pyrophosphatase MazG in *E. coli*. The significance of this complex is unknown; MazG does not influence the GTPase activity of Era (Zhang and Inouye, 2002). While MazG can degrade GTP, it cannot use pppGpp as substrate; however, MazG is under control of a (p)ppGpp-regulated promoter, and its overexpression significantly decreases (p)ppGpp levels during the stringent response (Aizenman et al., 1996; Gross et al., 2006).

### ERAL1 homeostasis in mammalian mitochondria

In mammalian cells, ERAL1 is subject to both negative and positive regulation by at least two distinct mechanisms (Figure 5C). It appears to be a target of two main mitochondrial ATP-dependent proteases, CLPP and LONP1 (Szczepanowska et al., 2016; Key et al., 2019). The connection between ERAL1 and CLPP is especially intriguing, since *CLPP* is also associated with the Perrault syndrome (Jenkinson et al., 2013). Knockout of *CLPP* had a dramatic impact on the mitochondrial ribosome, with a particularly strong effect on the SSU. Free SSUs were found to accumulate at the expense of monosomes, resulting in moderately decreased mitochondrial translation. Indeed, an analysis of potential CLPP substrates identified several mitoribosomal proteins and a few other factors involved in the mitoribosome biogenesis and translation, including ERAL1. *CLPP* knockout resulted in a significant stabilization and accumulation of ERAL1, accompanied by its higher recruitment to the SSU. It was hypothesized that the abnormally high level of SSU-bound ERAL1 prevents the recruitment of the r-protein bS1m and the subunit joining, and that CLPP could provide a degradative ERAL1 removal mechanism to enable the SSU assembly to proceed further. Indeed, dampening ERAL1 levels in CLPP-deficient cells somewhat improved mitochondrial translation (Szczepanowska et al., 2016). However, the molecular mechanism behind the CLPP deficiency is likely different. In contrast to bacterial bS1, which is a large, multidomain protein loosely bound at the very last stage of assembly, mitochondrial bS1m is a small integral component of the SSU which is already installed in the State A and is too far from ERAL1 to produce any steric clashes (Figures 4A,B). Furthermore, proteolytic degradation as an AF ejection mechanism is unknown, and it is difficult to envision how the very bulky CLPP or its hexameric cofactor CLPX could access ERAL1 in the spatially constrained SSU environment. CLPP more likely degrades ERAL1 outside the SSU. In our opinion, it is more plausible that by targeting ERAL1 and other SSU proteins CLPP maintains the balance between the two mitoribosomal subunits, preventing abnormal SSU overproduction which may be disruptive for translation (Bruni et al., 2021). This model is supported by the finding that uS7m, which is a key ERAL1 partner on the nascent SSU (Figures 4A, B) and another protein linked to the Perrault syndrome (Kline et al., 2022), is also a CLPP substrate (Szczepanowska et al., 2016).

More recently, a positive mechanism of ERAL1 control has been proposed (Reyes et al., 2020). The putative GTP/GDP-exchange factor RCC1L/WBSCR16 localizes to human mitochondria, and its shortest isoform, RCC1L^V3^, specifically interacts with the mitochondrial SSU. The RCCL1^V3^ pulldown robustly co-purified the GTPases ERAL1, NOA1, and mS29/DAP3. RCCL1^V3^ overexpression resulted in an increased recruitment of ERAL1 and NOA1 to the SSU and mildly impaired mitochondrial translation, echoing observations in CLPP-deficient cells (Szczepanowska et al., 2016). By contrast, RCCL1^V3^ knockdown significantly reduced the overall abundance of ERAL1 and the mitochondrial SSU and severely impacted the protein synthesis in the organelle, like it happened in the Perrault syndrome patient with the ERAL1^N236I^ mutation (Chatzispyrou et al., 2017). It is currently unclear whether RCCL1^V3^ is a genuine GEF for ERAL1 and/or other mitochondrial GTPases--such a mechanism would be an exciting twist in the biology of ribosome assembly-associated GTPases. But it does seem to be able to regulate the abundance and recruitment of ERAL1, likely at the level of the State A intermediate, where ERAL1, NOA1 and mS29 are all simultaneously present (Figure 4A).

The positive and negative regulatory mechanisms described in this section show that ERAL1 homeostasis is essential in mammalian cells as both ERAL1 deficiency and overproduction may be deleterious to mitochondrial translation, resulting in severe downstream effects, such as the Perrault syndrome.

## OPEN questions

After four decades of research, understanding of what and how Era does is still vague. This review attempted to make some order in disparate findings concerning Era across a wide variety of biological systems. This obviously brought new exciting questions awaiting experimental answers.

• What defines the presence and essentiality of Era in some organisms but not in others?
• What is the meaning, if any, of the persistent association of *era* with other conserved but translation-unrelated genes, such as *recO* and *cdd*?
• How do complex Era proteins (e.g., fused to YbeY and RNase III) function in the ribosome biogenesis?
• What is the actual working cycle of Era on the ribosome in function of its diverse ligands?
• When is Era recruited to the ribosome in different organisms: is there an earlier state where Era is not yet loaded?
• What exactly happens between the States B and C in the mitochondrial SSU assembly: what kind of events and factors enable all these tectonic changes and result in Era ejection?
• What are structural details of the Era-(p)ppGpp interaction?
• What is the destiny of under-assembled ribosomal subunits accumulating during the stringent response: can they be matured later or are simply degraded?
• Do Era proteins actually use GEFs, like RCC1L^V3^?
• How do apparently opposite molecular phenotypes, associated with *ERAL1*, *MRPS7* and *CLPP* mutations, result in so similar disease manifestations (Perrault syndrome)?
